# Lipid metabolism, BMI and the risk of nonalcoholic fatty liver disease in the general population: evidence from a mediation analysis

**DOI:** 10.1186/s12967-023-04047-0

**Published:** 2023-03-13

**Authors:** Song Lu, Qiyang Xie, Maobin Kuang, Chong Hu, Xinghui Li, Huijian Yang, Guotai Sheng, Guobo Xie, Yang Zou

**Affiliations:** 1 Department of Cardiology, Jiangxi Provincial People’s Hospital, Medical College of Nanchang University, Nanchang, 330006 Jiangxi China; 2 Jiangxi Cardiovascular Research Institute, Jiangxi Provincial People’s Hospital, The First Affiliated Hospital of Nanchang Medical College, Nanchang, 330006 Jiangxi China; 3 Department of Gastroenterology, Jiangxi Provincial People’s Hospital, The First Affiliated Hospital of Nanchang Medical College, Nanchang, 330006 Jiangxi China; 4 Fuzhou Dongxiang District People’s Hospital, Fuzhou, 331800 Jiangxi China

**Keywords:** BMI, Non-traditional lipid parameter, NAFLD, Mediation analysis, Remnant cholesterol

## Abstract

**Background:**

Body mass index (BMI) and lipid parameters are the most commonly used anthropometric parameters and biomarkers for assessing nonalcoholic fatty liver disease (NAFLD) risk. This study aimed to assess and quantify the mediating role of traditional and non-traditional lipid parameters on the association between BMI and NAFLD.

**Method:**

Using data from 14,251 subjects from the NAGALA (NAfld in the Gifu Area, Longitudinal Analysis) study, mediation analyses were performed to explore the roles of traditional [total cholesterol (TC), triglyceride (TG), high-density lipoprotein cholesterol (HDL-C), low-density lipoprotein cholesterol (LDL-C)] and non-traditional [non-HDL-C, remnant cholesterol (RC), TC/HDL-C ratio, LDL-C/HDL-C ratio, TG/HDL-C ratio, non-HDL-C/HDL-C ratio, and RC/HDL-C ratio] lipid parameters in the association of BMI with NAFLD and quantify the mediation effect of these lipid parameters on the association of BMI with NAFLD using the percentage of mediation.

**Result:**

After fully adjusting for confounders, multivariate regression analysis showed that both BMI and lipid parameters were associated with NAFLD (All *P-*value < 0.001). Mediation analysis showed that both traditional and non-traditional lipid parameters mediated the association between BMI and NAFLD (All *P*-value of proportion mediate < 0.001), among which non-traditional lipid parameters such as RC, RC/HDL-C ratio, non-HDL-C/HDL-C ratio, and TC/HDL-C ratio accounted for a relatively large proportion, 11.4%, 10.8%, 10.2%, and 10.2%, respectively. Further stratified analysis according to sex, age, and BMI showed that this mediation effect only existed in normal-weight (18.5 kg/m^2^ ≤ BMI < 25 kg/m^2^) people and young and middle-aged (30–59 years old) people; moreover, the mediation effects of all lipid parameters except TC accounted for a higher proportion in women than in men.

**Conclusion:**

The new findings of this study showed that all lipid parameters were involved in and mediated the risk of BMI-related NAFLD, and the contribution of non-traditional lipid parameters to the mediation effect of this association was higher than that of traditional lipid parameters, especially RC, RC/HDL-C ratio, non-HDL-C/HDL-C ratio, and TC/HDL-C ratio. Based on these results, we suggest that we should focus on monitoring non-traditional lipid parameters, especially RC and RC/HDL-C ratio, when BMI intervention is needed in the process of preventing or treating NAFLD.

**Supplementary Information:**

The online version contains supplementary material available at 10.1186/s12967-023-04047-0.

## Background

With the rapid economic growth, the changes in lifestyle, and the prevalence of obesity, the prevalence of NAFLD is increasing all over the world. It is estimated that about 1/4 of adults in the world have suffered from NAFLD [1–3], and in about ten years, the global prevalence of the disease is expected to further rise to 1/3 [4], which will bring huge public health problems and economic burden to society [5]. Therefore, it is necessary to carry out early prevention and risk factor screening for susceptible people for NAFLD.

High BMI and dyslipidemia are known to be the most important modifiable risk factors for the onset of NAFLD. A series of studies have elucidated the associations between BMI and dyslipidemia and NAFLD in the past, among which elevated body weight and dyslipidemia were the most important risk factors for the increased risk of NAFLD [6–14]. In addition, it is worth noting that with the rapid development of global industrialization and economy, many chemicals are released into the environment, and long-term to exposure of these pollutants will cause harm to human health, further leading to lipid metabolism disorders and weight gain [15, 16], which in turn further aggravates the disease burden of NAFLD. Toxicology-based evidence also supported this notion, with the most pronounced effect of increased chemical exposure on liver cells as a redox imbalance, often manifested in mitochondrial damage, enhancement of lipid peroxidation, elevation in reactive oxygen species formation, and depletion of intracellular reduced glutathione [17–21]. In the context of today's rapid global development, it is particularly important to be able to assess NAFLD risks more efficiently and accurately. Considering that the measurement of BMI is simple, while the inspection of lipid parameters is relatively complex, it may be a useful combination to use BMI as an indicator of daily NAFLD risk assessment and lipid parameters as indicators of regular NAFLD risk assessment; further clarification is needed which lipid parameters work best in combination with BMI for routine risk assessment of NAFLD. To address this issue, in the current study, we tried to explore the influence of all non-traditional and traditional lipid parameters on the association between BMI and NAFLD through the meditation analysis system.

## Method

### Data sources and study population

To elucidate the effect of lipid parameters on the association of BMI with NAFLD, we performed a secondary analysis using the NAGALA study dataset, which has been uploaded to the Dryad database for public share by the Okamura team (https://doi.org/10.5061/dryad.8q0p192). The NAGALA study, a population-based longitudinal cohort study, aimed at assessing risk factors for common chronic diseases, thereby reducing the burden on the public health system and promoting population health.

**Fig. 1:**
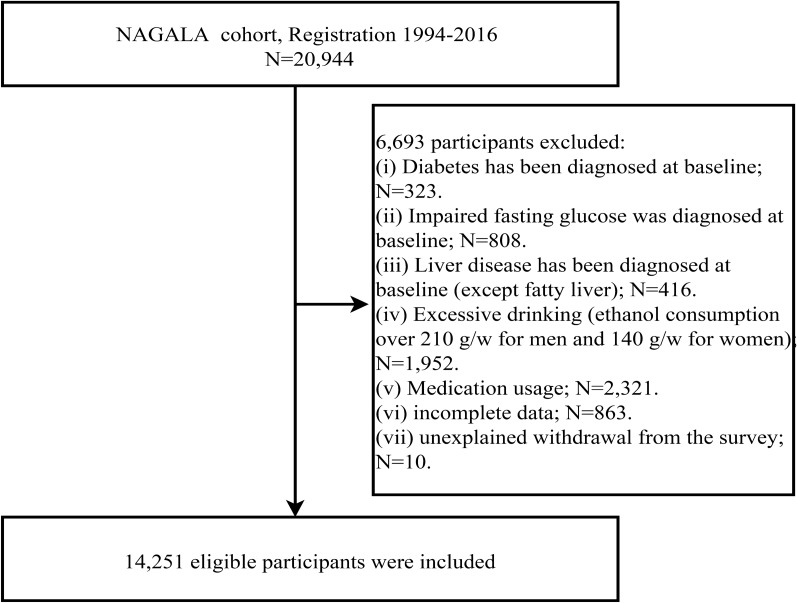
Flowchart of the selection process of study subjects

Detailed information about the NAGALA cohort study has been described in a previously published article [22]. Based on a new study objective, this study extracted physical examination data from 20,944 subjects in the NAGALA dataset between May 1994 and December 2016. According to the inclusion and exclusion criteria, we excluded subjects who were on medication at baseline, subjects with missing covariate data or excessive drinking or unexplained withdrawal from the study, and subjects with diabetes or impaired fasting glucose [fasting blood glucose (FPG) above 6.1 mmol/L] or liver disease (except fatty liver) at baseline. A total of 14,251 subjects were included in the final analysis of this study (Fig. 1).

### Ethics approval

The previous study was approved by the Murakami Memorial Hospital Ethics Committee [22], and written informed consent was obtained from each subject. The current study is a secondary analysis of the previous study, and the study protocol was approved by the Institutional Ethics Review Committee of Jiangxi Provincial People's Hospital, and the whole study process followed the Helsinki Declaration.

### Data collection and measurement

As mentioned earlier, standardized questionnaires were used to obtain subjects' age and sex, smoking and drinking status, the habit of exercise, disease history, and drug use history. Body weight, height, waist circumference (WC), and arterial blood pressure were professionally measured and recorded by professional medical workers. Where BMI was calculated as weight (kg)/[height(m)]^2^. A habit of exercise was defined as subjects participating in any form of physical activity more than one time per week. Drinking status was defined according to the amount of past weekly alcohol consumption of the subjects, and the subjects were classified as no or little drinkers, light drinkers, and moderate drinkers [23]. Smoking status was divided into three categories: none, former, and current. Blood specimens were collected from each subject after fasting for at least 8 h, and the following laboratory biochemical parameters, including gamma-glutamyl transferase (GGT), aspartate aminotransferase (AST), glycosylated hemoglobin (HbA1c), FPG and alanine aminotransferase (ALT), HDL-C, TC, and TG were analyzed and measured by an automated analyzer according to the standard method.

LDL-C and non-traditional lipid parameters are calculated as follows:

LDL-C (mg/dL) = 90%non-HDL-C – 10%TG [24];

Non-HDL-C = TC – HDL-C [25];

RC = non-HDL-C – LDL-C [26];

TC/HDL-C ratio = TC/HDL-C [27];

TG/HDL-C ratio = TG/HDL-C [28];

LDL-C/HDL-C ratio = LDL-C/HDL-C [29];

Non-HDL-C/HDL-C ratio = Non-HDL-C/HDL-C [30];

RC/HDL-C ratio = RC/HDL-C [31];

### Diagnosis of NAFLD

Trained professional technicians performed abdominal ultrasound examinations on the subjects. The ultrasound images were evaluated by experienced gastroenterologists without knowing the subjects' personal health data, and a final diagnosis was made based on a combination of four ultrasound sonogram features, including vascular blurring, liver and kidney echo contrast, depth attenuation and liver brightness [32].

### Statistical analysis

Descriptive data for baseline characteristics were expressed as mean (standard deviation) or median (interquartile range) for continuous variables and frequency (percentage) for categorical variables, and whether there were differences between groups were compared by chi-square test or Mann–Whitney U test or t test. In addition, we also calculated standardized difference values between the NAFLD group and non-NAFLD group to assess and quantify the magnitude of differences between groups (standardized difference values > 10% were considered significant), where skewed continuous variables were subjected to Box-COX normal transformation before data analysis [33, 34].

Before performing regression analysis and mediation analysis, we first evaluated the collinearity of lipid parameters/BMI and other covariates by multiple linear regression, and calculated the variance inflation factor as an evaluation tool, in which covariates with variance inflation factor greater than 10 were considered as collinear variable [35]. Collinearity screening results showed no high collinearity between all lipid parameters and baseline variables, but high collinearity between BMI and body weight (Additional file 1: Tables S1–S12). Based on these results of collinearity screening, we will exclude body weight in the subsequent regression analysis model and mediation analysis model, and further adjust the model according to epidemiological research evidence, and show the process of gradual adjustment in the main analysis [36].

We performed a mediation analysis according to the method suggested by Professor VanderWeele [37] to examine how lipid parameters as mediator variables affect the relationship between BMI (independent variable) and NAFLD (outcome variable). According to the requirements of the mediation analysis, the research data in this study must meet the following criteria: (1) BMI/lipid parameters must be associated with NAFLD: As a part of the study, we used multivariate logistic regression model to analyze the relationship between BMI/ lipid parameters and NAFLD, and considered the potential effects of sex, age, WC, habit of exercise, smoking status, drinking status, liver enzyme related factors (ALT, AST, GGT), blood pressure-related factors [systolic blood pressure (SBP), diastolic blood pressure (DBP)] and blood glucose metabolism factors (FPG, HbA1c) [1, 2, 38–40]; (2) BMI must be associated with lipid parameters: In this validation analysis, we assessed the association by multivariate linear regression in which we considered the potential effects of sex, age, WC, habit of exercise, smoking status, drinking status, blood pressure-related factors (SBP, DBP), and glucose metabolism factors (FPG, HbA1c) [2, 8, 40, 41]; (3) The association between BMI and NAFLD must to be attenuated when lipid parameters were included in the multivariate logistic regression models. If the above conditions are met then a mediation analysis can be performed to determine whether the effect of BMI on NAFLD is mediated by lipid parameters. In addition, the mediation percentage was obtained by calculating the ratio of the indirect effect to the total effect, thereby quantifying the size of the mediation effect. The significance of the mediation effect was tested using Bootstrap sampling (times = 1000) [42]. To reduce the effect of confounding factors, we adjusted for the covariates sex, age, WC, DBP, ALT, AST, GGT, FPG, SBP, HbA1c, the habit of exercise, smoking status, and drinking status in the mediation analysis [1, 2, 38–40]. Finally, we performed further exploratory stratified analyses for sex, age, and BMI, in which the age classification refers to the World Health Organization's classification standards (young people within 45 years old, middle-aged people between 45 and 59 years old, and elderly people over 60 years old), and the BMI classification refers to the World Health Organization's recommended BMI classification standards for Asian people [low weight (BMI < 18.5 kg/m^2^), normal weight (18.5 kg/m^2^ ≤ BMI < 25 kg/m^2^), and overweight/obese (BMI ≥ 25 kg/m^2^)] [43]. All analyses were done using R language version 3.4.3 and Empower(R) version 4. 0. All tests were two-tailed and statistical significance was set at *P* < 0.05.

## Result

### Characteristics of the study subjects

This study included 14,251 subjects with a mean age of 43.7 years, 48% of whom were women, and 17.6% of subjects were diagnosed with NAFLD. Subjects were grouped according to whether or not suffering from NAFLD, and Table 1 presents the differences in baseline characteristics of the study population. Except for drinking status, almost all baseline demographic data, anthropometric data, and serum biomarkers were significantly different between the NAFLD group and the non-NAFLD group (all *P* < 0.05). It is noteworthy that the BMI of the NAFLD group was significantly higher than that of the non-NAFLD group (25.5 vs 21.3), and the standardized difference value between the two groups was as high as 145%. In terms of lipid parameters, the standardized difference values between the two groups were generally higher for non-traditional lipid parameters than for traditional lipid parameters.

**Table 1:** Characteristics of the study subjects with and without NAFLD

	Non-NAFLD	NAFLD	Standardized difference (%)	*P-*value
No. of subjects	11,744	2507		
Sex			78 (74, 83)	< 0.001
Women	6362 (54.17%)	478 (19.07%)		< 0.001
Men	5382 (45.83%)	2029 (80.93%)		< 0.001
Age, years	42.00 (18.00–79.00)	44.00 (19.00–72.00)	18 (13, 22)	< 0.001
Weight, kg	57.72 (9.98)	72.18 (11.33)	135 (131, 140)	< 0.001
Height, cm	164.11 (8.44)	168.03 (7.90)	48 (44, 52)	< 0.001
BMI, kg/m^2^	21.33 (2.61)	25.50 (3.13)	145 (140, 149)	< 0.001
WC, cm	74.09 (7.92)	85.98 (7.79)	151 (147, 156)	< 0.001
ALT, U/L	15.00 (2.00–856.00)	27.00 (6.00–220.00)	96 (91, 100)	< 0.001
AST, U/L	17.00 (3.00–590.00)	20.00 (6.00–140.00)	56 (51, 60)	< 0.001
GGT, U/L	14.00 (3.00–259.00)	23.00 (6.00–375.00)	61 (57, 66)	< 0.001
TC, mmol/L	5.06 (0.85)	5.44 (0.87)	45 (41, 49)	< 0.001
TG, mmol/L	0.65 (0.07–10.27)	1.24 (0.16–7.69)	96 (91, 100)	< 0.001
HDL-C, mmol/L	1.52 (0.40)	1.19 (0.29)	96 (92, 101)	< 0.001
LDL-C. mmol/L	2.95 (0.95–9.72)	3.49 (1.16–6.59)	68 (64, 73)	< 0.001
Non-HDL-C, mmol/L	3.47 (1.22–10.93)	4.25 (1.38–7.71)	83 (79, 88)	< 0.001
RC, mmol/L	0.50 (0.17–2.72)	0.71 (0.18–2.37)	107 (102, 111)	< 0.001
TC/HDL-C ratio	3.33 (1.51–13.02)	4.71 (1.59–10.75)	111 (106, 115)	< 0.001
TG/HDL-C ratio	0.43 (0.03–16.55)	1.07 (0.12–11.67)	92 (87, 96)	< 0.001
LDL-C/HDL-C ratio	1.99 (0.43–10.35)	3.04 (0.50–8.01)	106 (101, 110)	< 0.001
Non-HDL-C/HDL-C ratio	2.33 (0.51–12.02)	3.71 (0.59–9.75)	111 (106, 115)	< 0.001
RC/HDL-C	0.33 (0.07–4.39)	0.62 (0.09–3.57)	107 (103, 112)	< 0.001
FPG, mmol/L	5.09 (0.40)	5.39 (0.36)	78 (74, 82)	< 0.001
HbA1c, %	5.15 (0.31)	5.30 (0.33)	46 (42, 51)	< 0.001
SBP, mmHg	111.91 (14.02)	123.41 (14.83)	80 (75, 84)	< 0.001
DBP, mmHg	69.69 (9.85)	77.81 (10.19)	81 (77, 85)	< 0.001
Habit of exercise	2093 (17.82%)	377 (15.04%)	8 (3, 12)	< 0.001
Drinking status			4 (-0.01, 9)	0.162
No or little	9717 (82.74%)	2088 (83.29%)		
Light	1472 (12.53%)	286 (11.41%)		
Moderate	555 (4.73%)	133 (5.31%)		
Smoking status			35 (31, 39)	< 0.001
None	7561 (64.38%)	1185 (47.27%)		
Former	1920 (16.35%)	639 (25.49%)		
Current	2263 (19.27%)	683 (27.24%)		

Values were expressed as mean (SD) or medians (quartile interval) or n (%). The differences between groups were compared by chi-square test or Mann–Whitney U test or t test (*P*-value < 0.05 indicates significance). Standardized difference values were used to evaluate and quantify the difference between groups (> 10% were considered significant)

NAFLD: Nonalcoholic fatty liver disease; BMI: body mass index; WC: Waist circumference; ALT: alanine aminotransferase; AST: aspartate aminotransferase; GGT: gamma-glutamyl transferase; HDL-C: high-density lipoprotein cholesterol; TC: total cholesterol; TG: triglyceride; LDL-C: low density lipoprotein cholesterol; Non-HDL-C: non-high-density lipoprotein cholesterol; RC: remnant cholesterol; HbA1c: hemoglobin A1c; FPG: fasting plasma glucose; SBP: systolic blood pressure; DBP: diastolic blood pressure

### Relationship between BMI/lipid parameters and NAFLD

After adjusting for potential confounders (Tables 2 and 3), there were significant associations between BMI or lipid parameters and NAFLD (all *P* < 0.001). The association between BMI and NAFLD was significantly positive in models 1–3 with Odds ratios (ORs) of 1.37 [95% confidence interval (CI)1.32–1.42, *P* < 0.001], 1.33 (1.29–1.38, *P* < 0.001), and 1.27 (1.22–1.31, *P* < 0.001), respectively; after further adjusting the lipid parameters (mediator variables), we found that the correlation between BMI and NAFLD weakened (Table 3, models 4–14), suggesting a mediation effect. In addition, with regard to lipid parameters, all lipid parameters were significantly positively associated with NAFLD except HDL-C. It is worth mentioning that the RC and the RC/HDL-C ratio were much riskier for NAFLD than other lipid parameters (RC: OR = 9.18, 95% CI 6.88–12.26, *P* < 0.001; RC/HDL-C ratio: OR = 5.01, 95% CI 4.02–6.24, *P* < 0.001).

**Table 2:** Relationship between lipid parameters and NAFLD

	OR (95% CI)		
	Model 1	Model 2	Model 3
BMI	1.37 (1.32, 1.42)*	1.33 (1.29, 1.38)*	1.27 (1.22, 1.31)*
TC	1.41 (1.33, 1.50)*	1.37 (1.29, 1.46)*	1.17 (1.09, 1.25)*
LDL-C	1.61 (1.50, 1.73)*	1.54 (1.43, 1.66)*	1.29 (1.19, 1.39)*
TG	2.27 (2.09, 2.47)*	2.25 (2.07, 2.45)*	1.93 (1.77, 2.11)*
HDL-C	0.25 (0.21, 0.31)*	0.25 (0.21, 0.31)*	0.30 (0.24, 0.36)*
Non-HDL-C	1.67 (1.57, 1.77)*	1.61 (1.51, 1.72)*	1.36 (1.27, 1.46)*
RC	18.53(14.17, 24.23)*	17.16(13.07, 22.54)*	9.18 (6.88, 12.26)*
TC/HDL-C ratio	1.57 (1.50, 1.65)*	1.56 (1.49, 1.64)*	1.40 (1.33, 1.47)*
TG/HDL-C ratio	1.94 (1.80, 2.08)*	1.92 (1.79, 2.07)*	1.68 (1.56, 1.82)*
LDL-C/HDL-C ratio	1.68 (1.58, 1.78)*	1.65 (1.56, 1.76)*	1.45 (1.36, 1.54)*
Non-HDL-C/HDL-C ratio	1.57 (1.50, 1.65*	1.56 (1.49, 1.64)*	1.40 (1.33, 1.47)*
RC/HDL-C ratio	7.94 (6.47, 9.74)*	7.79 (6.31, 9.62)*	5.01 (4.02, 6.24)*

OR: Odds ratios; CI: confidence interval; other abbreviations as in Table 1

^*^P < 0.001

Model 1 adjusted sex, age and WC

Model 2 adjusted model 1 + SBP, DBP, habit of exercise, smoking status and drinking status

Model 3 adjusted model 2 + ALT, AST, GGT, FPG and HbA1c

**Table 3:** Relationship between BMI and NAFLD

	OR (95% CI)	*P*-value
Model 1	1.37 (1.32, 1.42)	< 0.001
Model 2	1.33 (1.29, 1.38)	< 0.001
Model 3	1.27 (1.22, 1.31)	< 0.001
Model 4	1.26 (1.22, 1.31)	< 0.001
Model 5	1.24 (1.20, 1.29)	< 0.001
Model 6	1.25 (1.21, 1.30)	< 0.001
Model 7	1.26 (1.21, 1.31)	< 0.001
Model 8	1.25 (1.21, 1.30)	< 0.001
Model 9	1.25 (1.20, 1.30)	< 0.001
Model 10	1.24 (1.20, 1.29)	< 0.001
Model 11	1.25 (1.21, 1.30)	< 0.001
Model 12	1.25 (1.20, 1.29)	< 0.001
Model 13	1.24 (1.20, 1.29)	< 0.001
Model 14	1.25 (1.20, 1.29)	< 0.001

OR: Odds ratios; CI: confidence interval; other abbreviations as in Table 1

Model 1 adjusted sex, age and WC

Model 2 adjusted model I + SBP, DBP, habit of exercise, smoking status and drinking status

Model 3 adjusted model II + ALT, AST, GGT, FPG and HbA1c

Model 4 adjusted model II + TC; Model 5 adjusted model II + HDL-C; Model 6 adjusted model II + TG; Model 7 adjusted model II + LDL-C; Model 8 adjusted model II + non-HDL-C; Model 9 adjusted model II + RC; Model 10 adjusted model II + TC/HDL-C ratio; Model 11 adjusted model II + TG/HDL-C ratio; Model 12 adjusted model II + LDL-C/HDL-C ratio; Model 13 adjusted model II + non-HDL-C/HDL-C ratio; Model 14 adjusted model II + RC/HDL-C ratio

Models 4–14 show the correlation between BMI and NAFLD when lipid parameters are included in the regression model

### Relationship between BMI and lipid parameters

The results of multivariate linear regression models showed that all lipid parameters were significantly associated with BMI after fully adjusting for confounders (Additional file 1: Table S13); furthermore, it is worth mentioning that among all lipid parameters, RC and RC/HDL-C ratio were most positively associated with BMI (RC: β = 1.03, 95% CI 0.88–1.17, *P* < 0.001; RC/HDL-C ratio: β = 0.80, 95% CI 0.69–0.92, *P* < 0.001), while HDL-C was negatively associated with BMI.

### Mediation effect of lipid parameters on the association between BMI and NAFLD

**Fig. 2:**
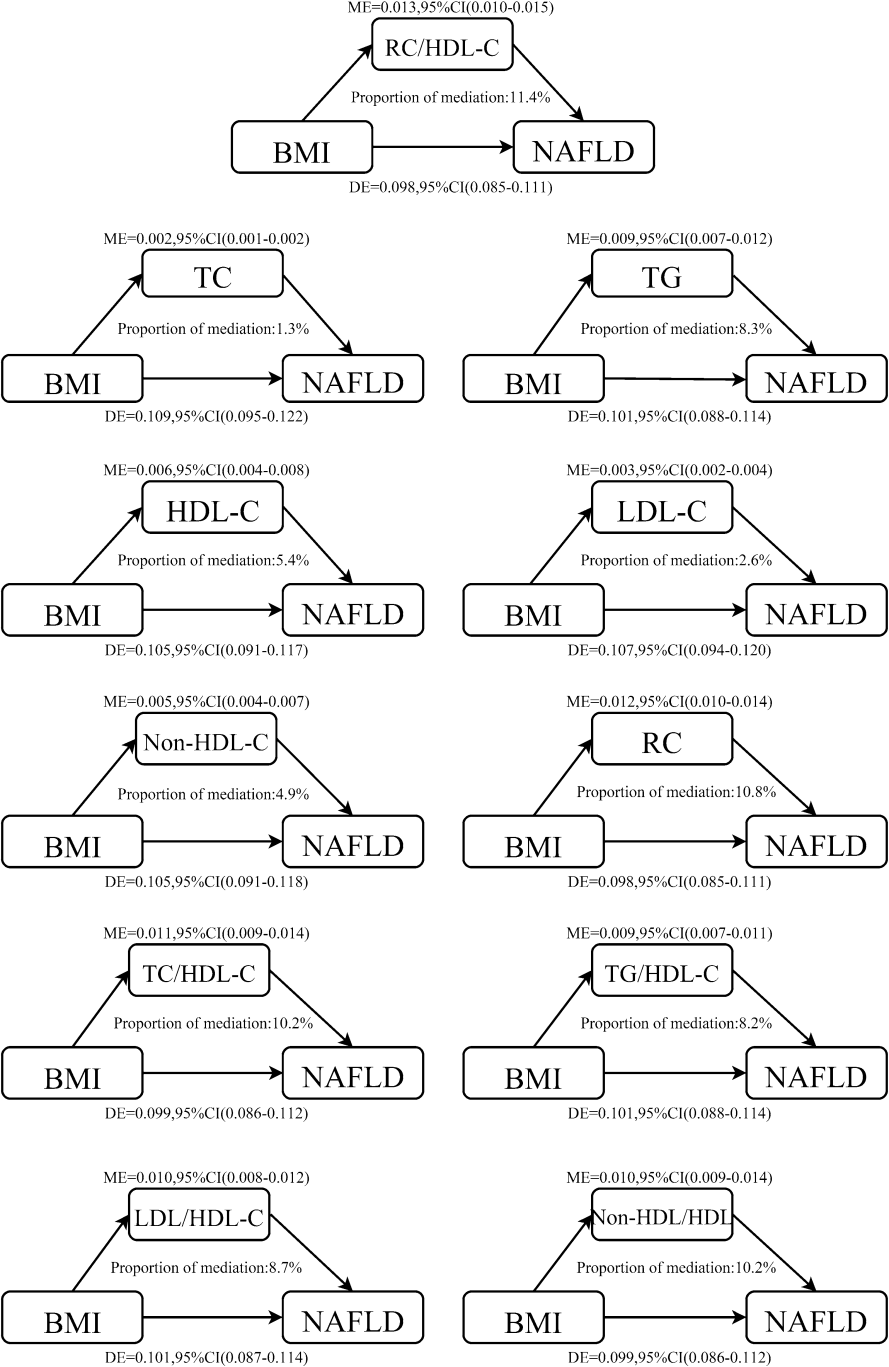
Lipid parameters mediation models of the relationship between BMI and NAFLD. ME: Mediation effect; DE: Direct effect; BMI: Body mass index; NAFLD: Nonalcoholic fatty liver disease

Table 4 shows the mediation analysis results of lipid parameters on the relationship between BMI and NAFLD. In the general population, we found that all 11 lipid parameters partially mediated the association between BMI and NAFLD risk, with RC/HDL-C ratio, RC, non-HDL-C/HDL-C ratio, and TC/HDL-C ratio mediating the largest mediation effects at 11.4%, 10.8%, 10.2%, and 10.2%, respectively (Fig. 2); while lipid parameters such as TC, HDL-C, LDL-C, and non-HDL-C played a weak role in mediating the association between BMI and NAFLD risk. Overall, non-traditional lipid parameters mediated the association between BMI and NAFLD more than traditional lipid parameters.

**Table 4:** Mediation analysis for BMI and NAFLD via lipid parameters in the whole population

Mediator	Total effect	Mediation effect	Direct effect	PM (%)	*P*-value of PM
TC	0.110 (0.097, 0.123)	0.002 (0.001, 0.002)	0.109 (0.095, 0.122)	1.3	< 0.001
TG	0.110 (0.097, 0.123)	0.009 (0.007, 0.012)	0.101 (0.088, 0.114)	8.3	< 0.001
HDL-C	0.110 (0.097, 0.123)	0.006 (0.004, 0.008)	0.105 (0.091, 0.117)	5.4	< 0.001
LDL-C	0.110 (0.097, 0.123)	0.003 (0.002, 0.004)	0.107 (0.094, 0.120)	2.6	< 0.001
Non-HDL-C	0.110 (0.097, 0.123)	0.005 (0.004, 0.007)	0.105 (0.091, 0.118)	4.9	< 0.001
RC	0.110 (0.097, 0.123)	0.012 (0.010, 0.014)	0.098 (0.085, 0.111)	10.8	< 0.001
TC/HDL-C ratio	0.110 (0.097, 0.123)	0.011 (0.009, 0.014)	0.099 (0.086, 0.112)	10.2	< 0.001
TG/HDL-C ratio	0.110 (0.097, 0.123)	0.009 (0.007, 0.011)	0.101 (0.088, 0.114)	8.2	< 0.001
LDL/HDL-C ratio	0.110 (0.097, 0.123)	0.010 (0.008, 0.012)	0.101 (0.087, 0.114)	8.7	< 0.001
Non-HDL-C/HDL-C ratio	0.110 (0.097, 0.123)	0.011 (0.009, 0.014)	0.099 (0.086, 0.112)	10.2	< 0.001
RC/HDL-C ratio	0.110 (0.097, 0.123)	0.013 (0.010, 0.015)	0.098 (0.085, 0.111)	11.4	< 0.001

PM: propotion mediate; other abbreviations as in Table 1

Adjusting variables: sex, age, WC, SBP, DBP, ALT, AST, GGT, FPG, HbA1c, habit of exercise, smoking status and drinking status

### Stratified analysis

We further stratified all subjects according to BMI, sex, and age, and explored the mediating role of lipid parameters in the association between BMI and NAFLD in different populations.

Table 5 shows the mediation effect of lipid parameters on the association between BMI and NAFLD in different BMI groups. The results showed that the mediation effect of lipid parameters on the association between BMI and NAFLD was only observed in the normal-weight group, while no significant mediation effect was found in the low-weight group and the overweight/obese group. In general, in people with normal BMI, the mediation effect of non-traditional lipid parameters was greater than that of traditional lipid parameters, among which the meditation percentages of non-traditional lipid parameters such as RC/HDL-C ratio, RC, TC/HDL-C ratio, non-HDL-C/HDL-C ratio, LDL-C/HDL-C ratio, TG/HDL-C ratio were 17.2%, 16.2%, 15.9%, 15.9%, 13.9%, and 12.2%, respectively.

**Table 5:** Mediation analysis for BMI and NAFLD via lipid parameters in the whole population stratified by BMI

Mediator	Total effect	Mediation effect	Direct effect	PM (%)	*P*-value of PM
BMI < 18.5 kg/m^2^ (n = 1545)					
TC	− 0.000 (− 0.004, 0.003)	0.000 (− 0.000, 0.000)	− 0.001 (− 0.004, 0.003)	–	0.912
TG	− 0.000 (− 0.004, 0.003)	0.000 (− 0.000, 0.000)	− 0.001 (− 0.004, 0.003)	–	0.878
HDL-C	− 0.000 (− 0.004, 0.003)	− 0.000 (− 0.000, 0.000)	− 0.000 (− 0.004, 0.003)	–	0.910
LDL-C	− 0.000 (− 0.004, 0.003)	0.000 (− 0.000, 0.000)	− 0.001 (− 0.004, 0.003)	–	0.878
Non-HDL-C	− 0.000 (− 0.004, 0.003)	0.000 (− 0.000, 0.000)	− 0.001 (− 0.004, 0.003)	–	0.858
RC	− 0.000 (− 0.004, 0.003)	0.000 (− 0.000, 0.001)	− 0.001 (− 0.004, 0.003)	–	0.854
TC/HDL-C ratio	− 0.000 (− 0.004, 0.003)	0.000 (− 0.000, 0.000)	− 0.000 (− 0.004, 0.003)	–	0.974
TG/HDL-C ratio	− 0.000 (− 0.004, 0.003)	0.000 (− 0.000, 0.000)	− 0.000 (− 0.004, 0.003)	–	0.970
LDL/HDL-C ratio	− 0.000 (− 0.004, 0.003)	0.000 (− 0.000, 0.000)	− 0.000 (− 0.004, 0.003)	–	0.966
Non-HDL-C/HDL-C ratio	− 0.000 (− 0.004, 0.003)	0.000 (− 0.000, 0.000)	− 0.000 (− 0.004, 0.003)	–	0.974
RC/HDL-C ratio	− 0.000 (− 0.004, 0.003)	0.000 (− 0.000, 0.000)	− 0.000 (− 0.004, 0.003)	–	0.972
BMI: 18.5–25 kg/m^2^ (n = 10,442)					
TC	0.062 (0.048, 0.076)	0.001 (0.001, 0.002)	0.060 (0.046, 0.074)	2.4	< 0.001
TG	0.062 (0.048, 0.076)	0.007 (0.005, 0.010)	0.054 (0.040, 0.068)	12.1	< 0.001
HDL-C	0.062 (0.048, 0.076)	0.005 (0.004, 0.007)	0.057 (0.043, 0.071)	8.3	< 0.001
LDL-C	0.062 (0.048, 0.076)	0.003 (0.002, 0.005)	0.059 (0.045, 0.073)	5.1	< 0.001
Non-HDL-C	0.062 (0.048, 0.076)	0.005 (0.004, 0.007)	0.057 (0.043, 0.071)	8.3	< 0.001
RC	0.062 (0.048, 0.076)	0.010 (0.008, 0.012)	0.052 (0.038, 0.066)	16.2	< 0.001
TC/HDL-C ratio	0.062 (0.048, 0.076)	0.010 (0.008, 0.012)	0.052 (0.038, 0.066)	15.9	< 0.001
TG/HDL-C ratio	0.062 (0.048, 0.076)	0.008 (0.005, 0.010)	0.054 (0.040, 0.068)	12.2	< 0.001
LDL/HDL-C ratio	0.062 (0.048, 0.076)	0.009 (0.007, 0.011)	0.053 (0.039, 0.067)	13.9	< 0.001
Non-HDL-C/HDL-C ratio	0.062 (0.048, 0.076)	0.010 (0.008, 0.012)	0.052 (0.038, 0.066)	15.9	< 0.001
RC/HDL-C ratio	0.062 (0.048, 0.076)	0.011 (0.008, 0.013)	0.051 (0.037, 0.065)	17.2	< 0.001
BMI: ≥ 25 kg/m^2^ (n = 2264)					
TC	0.078 (0.045, 0.113)	0.000 (− 0.001, 0.002)	0.077 (0.045, 0.113)	–	0.826
TG	0.077 (0.044, 0.113)	− 0.002 (− 0.009, 0.004)	0.080 (0.047, 0.113)	–	0.478
HDL-C	0.077 (0.045, 0.111)	0.002 (− 0.003, 0.008)	0.075 (0.043, 0.110)	–	0.410
LDL-C	0.077 (0.044, 0.113)	0.000 (− 0.001, 0.002)	0.077 (0.044, 0.112)	–	0.558
Non-HDL-C	0.077 (0.044, 0.112)	0.001 (− 0.002, 0.004)	0.077 (0.044, 0.112)	–	0.666
RC	0.077 (0.044, 0.113)	− 0.001(− 0.007, 0.005)	0.079 (0.046, 0.112)	–	0.712
TC/HDL-C ratio	0.077 (0.043, 0.112)	0.001 (− 0.003, 0.007)	0.075 (0.043, 0.109)	–	0.556
TG/HDL-C ratio	0.077 (0.043, 0.112)	− 0.002 (− 0.008, 0.005)	0.078 (0.046, 0.112)	–	0.676
LDL/HDL-C ratio	0.077 (0.043, 0.111)	0.002 (− 0.002, 0.006)	0.075 (0.042, 0.109)	–	0.442
Non-HDL-C/HDL-C ratio	0.077 (0.043, 0.112)	0.001 (− 0.003, 0.007)	0.075 (0.043, 0.109)	–	0.556
RC/HDL-C ratio	0.077 (0.043, 0.112)	− 0.000 (− 0.006, 0.006)	0.077 (0.045, 0.111)	–	0.930

PM: propotion mediate; other abbreviations as in Table 1

Adjusting variables: sex, age, WC, SBP, DBP, ALT, AST, GGT, FPG, HbA1c, habit of exercise, smoking status and drinking status

In the mediation analysis stratified by sex (Table 6), we found that the mediation effect of lipid parameters on the association between BMI and NAFLD was generally higher in women than in men; among them, the mediation percentages of lipid parameters such as RC/HDL-C ratio, RC, TG/HDL-C ratio, TG, TC/HDL-C ratio and non-HDL-C/HDL-C ratio were more than 10% in women (15.4%, 12.9%, 12.5%, 11.7%, 11.5%, 11.5%, respectively, *P* < 0.001). While the mediation effect of the RC/HDL-C ratio was the largest in men, accounting for 7%.

**Table 6:** Mediation analysis for BMI and NAFLD via lipid parameters in the whole population stratified by sex

Mediator	Total effect	Mediation effect	Direct effect	PM (%)	*P*-value of PM
Men (n = 7441)					
TC	0.110 (0.088, 0.132)	0.001 (0.000, 0.002)	0.109 (0.087, 0.131)	1	0.006
TG	0.110 (0.087, 0.132)	0.005 (0.001, 0.008)	0.105 (0.083, 0.128)	4.4	< 0.001
HDL-C	0.111 (0.088, 0.133)	0.004 (0.002, 0.007)	0.106 (0.084, 0.128)	3.9	< 0.001
LDL-C	0.110 (0.088, 0.132)	0.002 (0.001, 0.003)	0.108 (0.086, 0.131)	1.7	< 0.001
Non-HDL-C	0.110 (0.087, 0.132)	0.004 (0.002, 0.006)	0.107 (0.084, 0.129)	3.2	< 0.001
RC	0.110 (0.087, 0.132)	0.007 (0.003, 0.010)	0.104 (0.081, 0.126)	5.9	< 0.001
TC/HDL-C ratio	0.111 (0.088, 0.133)	0.008 (0.005, 0.011)	0.103 (0.080, 0.125)	7	< 0.001
TG/HDL-C ratio	0.111 (0.088, 0.132)	0.006 (0.003, 0.010)	0.105 (0.082, 0.127)	5.5	< 0.001
LDL/HDL-C ratio	0.111 (0.088, 0.132)	0.007 (0.004, 0.009)	0.104 (0.082, 0.127)	5.9	< 0.001
Non-HDL-C/HDL-C ratio	0.111 (0.088, 0.133)	0.008 (0.005, 0.011)	0.103 (0.080, 0.125)	7	< 0.001
RC/HDL-C ratio	0.111 (0.088, 0.132)	0.008 (0.005, 0.012)	0.102 (0.080, 0.125)	7	< 0.001
Women (n = 6840)					
TC	0.084 (0.073, 0.097)	0.001 (0.000, 0.002)	0.083 (0.072, 0.096)	1	0.046
TG	0.084 (0.073, 0.096)	0.010 (0.008, 0.012)	0.074 (0.063, 0.087)	11.7	< 0.001
HDL-C	0.084 (0.073, 0.097)	0.005 (0.003, 0.006)	0.080 (0.069, 0.092)	5.4	< 0.001
LDL-C	0.084 (0.073, 0.097)	0.003 (0.001, 0.004)	0.082 (0.070, 0.094)	3.1	0.002
Non-HDL-C	0.084 (0.073, 0.096)	0.004 (0.002, 0.006)	0.080 (0.069, 0.093)	4.9	< 0.001
RC	0.084 (0.073, 0.096)	0.011 (0.009, 0.013)	0.073 (0.062, 0.086)	12.9	< 0.001
TC/HDL-C ratio	0.084 (0.073, 0.096)	0.010 (0.007, 0.012)	0.075 (0.063, 0.087)	11.5	< 0.001
TG/HDL-C ratio	0.084 (0.073, 0.096)	0.011 (0.008, 0.013)	0.074 (0.062, 0.086)	12.5	< 0.001
LDL/HDL-C ratio	0.084 (0.073, 0.096)	0.008 (0.006, 0.011)	0.076 (0.064, 0.089)	9.8	< 0.001
Non-HDL-C/HDL-C ratio	0.084 (0.073, 0.096)	0.010 (0.007, 0.012)	0.075 (0.063, 0.087)	11.5	< 0.001
RC/HDL-C ratio	0.084 (0.073, 0.096)	0.013 (0.010, 0.016)	0.071 (0.060, 0.084)	15.4	< 0.001

PM: propotion mediate; other abbreviations as in Table 1

Adjusting variables: age, WC, SBP, DBP, ALT, AST, GGT, FPG, HbA1c, habit of exercise, smoking status and drinking status

In the mediation analysis stratified by age (Additional file 1: Table S14), we only found significant mediation effects in the 30–44 years old and 45–59 years old subgroups, and the results were basically consistent with the results of the whole population. The contribution of non-traditional lipid parameters to the risk of NAFLD associated with BMI was higher than that of traditional lipid parameters, especially RC, RC/HDL-C ratio, non-HDL-C/HDL-C ratio, and TC/HDL-C ratio.

## Discussion

In this data analysis of 14,251 general subjects based on the NAGALA cohort, we have found that both traditional and non-traditional lipid parameters played an important role in the association between BMI and NAFLD, among which the non-traditional lipid parameters RC/HDL-C ratio, RC, non-HDL-C/HDL-C ratio and TC/HDL-C ratio accounted for a large proportion in mediating the association between BMI and NAFLD, which were 11.4%, 10.8%, 10.2%, and 10.2%, respectively. The further stratified analysis also found that this mediation effect existed only in normal-weight people and young and middle-aged people (30–59 years old); in addition, compared with men, all lipid parameters except TC accounted for a higher proportion of mediation effects in women.

NAFLD has become a global health problem, affecting 1/4 of the global population, and its rising prevalence is synchronized with the prevalence of metabolic syndrome, obesity, and type 2 diabetes [3]. In addition, a large number of epidemiological evidence showed that BMI and dyslipidemia were closely related to the occurrence and development of NAFLD [8, 11–14, 44]. NAFLD is a complex metabolic-related disease, and the latest research has proposed to change the name of NAFLD to metabolic-associated fatty liver disease [45] because this name was more in line with the pathophysiological process of NAFLD. Early management of these controllable risk factors for metabolic disorders may be useful and efficient in reducing the risk of NAFLD [46].

Similar to the results of some previous studies [11–14, 47–52], we have also reported the relationship between BMI and lipid parameters and NAFLD in the current study. The results showed that there was a significant correlation between BMI and lipid parameters and NAFLD after adjusting for potential confounding factors. In addition, after further adjustment of lipid parameters, we observed that the magnitude of the association between BMI and NAFLD had decreased, which suggested that lipid parameters may play a role in mediating the association, thereby supporting the next step of mediation analysis in this study. At present, little is known about the relationship between BMI and NAFLD mediated by lipid parameters. As far as we know, there is only one study to explore the relationship between BMI and NAFLD mediated by TG [53]. In the study by Xing et al., who analyzed data from 15,943 employees from the Kailuan Group in China, they showed that approximately 26% of the effect of BMI on NAFLD was mediated through TG levels in the high BMI group (BMI ≥ 24 kg/m^2^), while in the low BMI group (BMI < 24 kg/m^2^), no significant mediation effect was observed. In this study, we evaluated the meditation percentage of TG-mediated BMI and NAFLD association in the whole population to be 8.3%, while further stratification according to the cut-off point of BMI in the Asian population, we found that the mediation effect of TG-mediated BMI and NAFLD association was only observed in normal-weight people, but it was not significant in low-weight people and overweight/obese people. In order to explore whether the inconsistency between the research results of Xing et al. and the current research results is related to the difference in BMI cut-off points, we continued to use the same BMI cut-off points as that of Xing et al. for further exploratory analysis [53]. The results still showed that the meditation effect of TG on the association between BMI and NAFLD existed only in normal-weight people (Additional file 1: Table S15). On the reasons why the results remain inconsistent after further analysis, we considered that it may be related to the different constituent ratios of the study population. In the current study, overweight/obese people only accounted for 15.89%, even if using 24 kg/m^2^ as the BMI cut-off point, the proportion of overweight/obese people in our study was only 23.65%, but in the study of Xing et al., the proportion of overweight/obesity people was 45.78% [53]. In addition, it is worth mentioning that Xing et al. studied coal miners with an average age of 51 years old in northern China (average TG was 1.10 mmol/L), while our subjects were general people with an average age of 43 years (average TG was 0.89 mmol/L). It is well known that coal workers have been exposed to a large amount of coal dust for a long time, which can lead to many health problems [54], and the most common of which are lung disease and heart disease (arrhythmia and myocardial ischemia). Coal dust contains a variety of toxic substances, including acrolein, which can have a serious impact on lipid metabolism, especially glycerol phospholipid metabolism [55–57]. In further animal-based studies, Gasparotto et al. also found that coal dust can further enhance the pro-inflammatory characteristics of obese rats and induce microvesicular steatosis [58]. Correlative evidence based on toxicology studies further suggested that hepatotoxicity induced by increased chemical exposure may be the result of oxidative hazards that lead to mitochondrial/lysosomal toxic connection and disorders in biochemical markers, and antioxidants can play an important role in reversing this process [17–21]. Some clinical evidence also supported this important discovery [59, 60]. These findings may explain the higher proportion of TG-mediated BMI and NAFLD association in the study by Xing et al., and provide useful data for the prevention and treatment of NAFLD. Considering the uniqueness of the human body, combined with the existing research results based on human samples, we suggested that people at high risk of NAFLD should pay attention to increasing the intake of natural antioxidants and trace element antioxidants from dietary sources at an early stage, such as anthocyanins, curcumin and resveratrol as well as coffee, tea, soy, vitamin C, vitamin E and astaxanthin [2, 59, 61, 62]. For NAFLD patients with dyslipidemia or high-risk groups, it is recommended to use antioxidant vitamin C and vitamin E in combination on the basis of statin therapy. The recommended dose is 20 mg of atorvastatin, 1 g of vitamin C and 1000 IU of vitamin E daily mix of [60]. In addition, therapeutic drugs acting on hepatocyte channels should also be paid attention to, because dysfunctional gap junctions are closely related to the pathogenesis of NAFLD, and normally functioning channels contribute to the maintenance of tissue homeostasis and liver function [63].

In addition to the BMI subgroup, we also conducted the same analysis in the sex subgroup and age subgroup. In general, even after further stratification, we can still observe that the contribution of non-traditional lipid parameters to the association of BMI with NAFLD is higher than that of traditional lipid parameters in subgroups. Similar findings has been reported in several previous studies where non-traditional lipid parameters had better value than traditional lipid parameters in the assessment of the risk of NAFLD onset/prevalence [25–31, 64]. These new findings may provide targeted monitoring recommendations for BMI intervention in different populations. In the current study, we also found that almost all lipid parameters had a higher mediation effect on BMI and NAFLD association in women than in men, and the two non-traditional lipid parameters, RC/HDL-C ratio and RC, were the most noteworthy, which played a more important role in BMI and NAFLD association than other lipid parameters. The reason for this special phenomenon is currently unknown, and it may be related to the function of BMI-related but obviously sex-specific substances in the body. Based on a recent targeted lipidomic analysis of 10,339 adults in the AusDiab study, Beyene et al. measured 706 lipids from 36 different lipid classes and assessed whether the relationship between BMI and lipidomic profiles differed by sex. The results showed that acylcarnitine species exhibited opposite associations in men and women [65], a finding that may provide a potential direction to further explore the findings of the current study. According to the findings of the current study, we recommend that women should focus on monitoring non-traditional lipid parameters, especially RC/HDL-C ratio and RC if they need to prevent or treat NAFLD through the intervention of modifiable risk factors. Moreover, we further evaluated the effect of lipid parameters on BMI and NAFLD association in different age groups, and finally found significant mediation effects in 30–44 years old and 45–59 years old subgroups. The results also supported that the mediation effects of non-traditional lipid parameters in BMI and NAFLD association were greater than that of traditional lipid parameters, in which RC/HDL-C ratio and RC played a key role in mediating the association in people aged 30–44, while in 45–59 years old, only RC/HDL-C ratio played a role in mediating more than 10% of the association. Overall, regardless of sex, age, fatness or thinness, we recommend that at least attention should be paid to the monitoring of the RC/HDL-C ratio, a non-traditional lipid parameter.

The mechanism of BMI and NAFLD association mediated by lipid parameters is still unclear. Given the relationship between BMI and lipid parameters and NAFLD, we speculated that insulin resistance (IR) may have an important influence on this mediation effect [8, 11–14]. Additionally, the findings of the current study may be of great significance for understanding how BMI leads to the onset of NAFLD and provide new and useful references for NAFLD risk intervention. At present, some studies have described the evidence that intervention against both BMI and dyslipidemia can reduce the risk of NAFLD. In the study of Magkos et al. [66], they found that progressive weight loss will improve human adipose disease and IR, and weight loss of 5%, 11%, and 16% can reduce TG levels in the liver by 13%, 52%, and 65%, respectively; not only that, serum TG levels also showed a significant downward trend. Moreover, Promrat and Lazo et al. also had similar findings that weight loss will greatly improve liver steatosis [67, 68]. The above findings were very useful, and based on the results of this study, we suggested that we also need to strengthen the monitoring of non-traditional lipid parameters, especially the RC/HDL-C ratio and RC levels, which may be more valuable simple parameters that can improve compliance and success rate for health behavior changes. For future work, we suggested: (1) Further follow-up studies to verify the effectiveness of monitoring traditional/non-traditional lipid parameters in different populations for future NAFLD risk assessment. (2) Further research on drug intervention, including antioxidants, lipid-lowering drugs and cell channel modification drugs. (3) Medical workers or public health decision makers incorporate the joint management of BMI and lipid parameters into the disease prevention or treatment programs of NAFLD, and special attentions should be paid to the monitoring role of unconventional lipid parameters.

### Limitations

There were several limitations in the current study that need to be acknowledged: (1) Lipid parameters explained only part of the association between BMI and NAFLD, and further studies are needed to evaluate other potential mediators that mediate the association between BMI and NAFLD. (2) The study subjects were the Japanese general population, and BMI was classified according to the criteria suitable for Asians, so the results of this study should be used with caution for other ethnic groups. (3) Serum insulin was not measured in this study, and IR status could not be assessed, so the important role of IR in the association could not be further clarified. (4) The mechanisms behind sex and age affecting the mediation effects of lipid parameters remain unclear, and further longitudinal studies are needed to clarify the potential associations and effects. (5) NAFLD was not graded in the current study, so it was not possible to further assess whether the association of BMI with different grades of NAFLD was mediated by lipid parameters. (6) Although we have excluded the subjects who are taking drugs at baseline at the beginning of the study, and adjusted the factors including exercise habits, smoking and drinking status, blood pressure, blood glucose, liver enzyme metabolism, sex, age and WC in the subsequent data analysis, it is undeniable that there are still many life-related factors that have not been considered in the current study, which will inevitably lead to some residual confounding.

## Conclusion

In conclusion, all lipid parameters were found to be involved in and mediate the risk of NAFLD associated with BMI in this study, with non-traditional lipid parameters contributing more to the mediation effect of the association than traditional lipid parameters, especially RC, RC/HDL-C ratio, non-HDL-C/HDL-C ratio, and TC/HDL-C ratio; These findings provided new ideas for the prevention and treatment of NAFLD, we call for more attention to non-traditional lipid parameters when intervening on BMI as a controllable risk factor.

## Supplementary Information

**Additional file 1: Table S1.** Collinearity diagnostics steps of TC with other covariates. **Table S2.** Collinearity diagnostics steps of HDL-C with other covariates. **Table S3.** Collinearity diagnostics steps of TG with other covariates. **Table S4.** Collinearity diagnostics steps of LDL-C with other covariates. **Table S5.** Collinearity diagnostics steps of non-HDL-C with other covariates. **Table S6.** Collinearity diagnostics steps of RC with other covariates. **Table S7.** Collinearity diagnostics steps of TC/HDL-C ratio with other covariates. **Table S8.** Collinearity diagnostics steps of TG/HDL-C ratio with other covariates. **Table S9.** Collinearity diagnostics steps of LDL/HDL-C ratio with other covariates. **Table S10.** Collinearity diagnostics steps of non-HDL/HDL-C ratio with other covariates. **Table S11.** Collinearity diagnostics steps of RC/HDL-C ratio with other covariates. **Table S12.** Collinearity diagnostics steps of BMI with other covariates. **Table S13.** Association of BMI with lipid parameters. **Table S14.** Mediation analysis for BMI and NAFLD via lipid parameters in the whole population stratified by age. **Table S15.** Mediation analysis for BMI and NAFLD via lipid parameters in the whole population stratified by BMI.

## Data Availability

The data used in this study have been uploaded to the "Dryad" database by Professor Okamura et al. (https://doi.org/10.5061/dryad.8q0p192).

## Abbreviations

TC: Total cholesterol
TG: Triglyceride
HDL-C: High-density lipoprotein cholesterol
LDL-C: Low-density lipoprotein cholesterol
RC: Remnant cholesterol
BMI: Body mass index
AST: Aspartate aminotransferase
GGT: Gamma-glutamyl transferase
ALT: Alanine aminotransferase
FPG: Fasting plasma glucose
HbA1c: Hemoglobin A1c
NAGALA: NAfld in the Gifu Area, Longitudinal Analysis
IR: Insulin resistance
ORs: Odds ratios
CI: Confidence interval
NAFLD: Nonalcoholic fatty liver disease

## References

[1] Milić S, Lulić D, Štimac D (2014). Non-alcoholic fatty liver disease and obesity: biochemical, metabolic and clinical presentations. World J Gastroenterol.

[2] Hernandez-Rodas MC, Valenzuela R, Videla LA (2015). Relevant aspects of nutritional and dietary interventions in non-alcoholic fatty liver disease. Int J Mol Sci.

[3] Younossi Z, Anstee QM, Marietti M, Hardy T, Henry L, Eslam M (2018). Global burden of NAFLD and NASH: trends, predictions, risk factors and prevention. Nat Rev Gastroenterol Hepatol.

[4] Estes C, Razavi H, Loomba R, Younossi Z, Sanyal AJ (2018). Modeling the epidemic of nonalcoholic fatty liver disease demonstrates an exponential increase in burden of disease. Hepatology.

[5] Younossi ZM, Blissett D, Blissett R, Henry L, Stepanova M, Younossi Y (2016). The economic and clinical burden of nonalcoholic fatty liver disease in the United States and Europe. Hepatology.

[6] Soares ALG, Banda L, Amberbir A, Jaffar S, Musicha C, Price AJ (2020). A comparison of the associations between adiposity and lipids in Malawi and the United Kingdom. BMC Med.

[7] Musso G, Cassader M, Paschetta E, Gambino R (2018). Bioactive lipid species and metabolic pathways in progression and resolution of nonalcoholic steatohepatitis. Gastroenterology.

[8] Katsiki N, Mikhailidis DP, Mantzoros CS (2016). Non-alcoholic fatty liver disease and dyslipidemia: an update. Metabolism.

[9] Gaggini M, Morelli M, Buzzigoli E, DeFronzo RA, Bugianesi E, Gastaldelli A (2013). Non-alcoholic fatty liver disease (NAFLD) and its connection with insulin resistance, dyslipidemia, atherosclerosis and coronary heart disease. Nutrients.

[10] Speliotes EK, Massaro JM, Hoffmann U, Vasan RS, Meigs JB, Sahani DV (2010). Fatty liver is associated with dyslipidemia and dysglycemia independent of visceral fat: the Framingham Heart Study. Hepatology.

[11] Chen TP, Lin WY, Chiang CH, Shen TH, Huang KC, Yang KC (2021). Metabolically healthy obesity and risk of non-alcoholic fatty liver disease severity independent of visceral fat. J Gastroenterol Hepatol.

[12] Lonardo A, Mantovani A, Lugari S, Targher G (2020). Epidemiology and pathophysiology of the association between NAFLD and metabolically healthy or metabolically unhealthy obesity. Ann Hepatol.

[13] Tang Z, Pham M, Hao Y, Wang F, Patel D, Jean-Baptiste L (2019). Sex, Age, and BMI modulate the association of physical examinations and blood biochemistry parameters and NAFLD: a retrospective study on 1994 cases observed at Shuguang Hospital. China Biomed Res Int.

[14] Cho HC (2016). Prevalence and factors associated with nonalcoholic fatty liver disease in a nonobese Korean Population. Gut Liver.

[15] Zheng S, Yang Y, Wen C, Liu W, Cao L, Feng X (2021). Effects of environmental contaminants in water resources on nonalcoholic fatty liver disease. Environ Int.

[16] Thayer KA, Heindel JJ, Bucher JR, Gallo MA (2012). Role of environmental chemicals in diabetes and obesity: a National Toxicology Program workshop review. Environ Health Perspect.

[17] Videla LA, Valenzuela R (2022). Perspectives in liver redox imbalance: toxicological and pharmacological aspects underlying iron overloading, nonalcoholic fatty liver disease, and thyroid hormone action. BioFactors.

[18] Ahmadian E, Eftekhari A, Fard JK, Babaei H, Nayebi AM, Mohammadnejad D (2017). In vitro and in vivo evaluation of the mechanisms of citalopram-induced hepatotoxicity. Arch Pharm Res.

[19] Ahmadian E, Babaei H, Mohajjel Nayebi A, Eftekhari A, Eghbal MA (2017). Mechanistic approach for toxic effects of bupropion in primary rat hepatocytes. Drug Res.

[20] Eftekhari A, Hasanzadeh A, Khalilov R, Hosainzadegan H, Ahmadian E, Eghbal MA (2020). Hepatoprotective role of berberine against paraquat-induced liver toxicity in rat. Environ Sci Pollut Res Int.

[21] Eghbal MA, Taziki S, Sattari MR (2014). Mechanisms of phenytoin-induced toxicity in freshly isolated rat hepatocytes and the protective effects of taurine and/or melatonin. J Biochem Mol Toxicol.

[22] Okamura T, Hashimoto Y, Hamaguchi M, Obora A, Kojima T, Fukui M (2019). Ectopic fat obesity presents the greatest risk for incident type 2 diabetes: a population-based longitudinal study. Int J Obes (Lond).

[23] Hashimoto Y, Hamaguchi M, Kojima T, Ohshima Y, Ohbora A, Kato T (2015). Modest alcohol consumption reduces the incidence of fatty liver in men: a population-based large-scale cohort study. J Gastroenterol Hepatol.

[24] Chen Y, Zhang X, Pan B, Jin X, Yao H, Chen B (2010). A modified formula for calculating low-density lipoprotein cholesterol values. Lipids Health Dis.

[25] Zelber-Sagi S, Salomone F, Yeshua H, Lotan R, Webb M, Halpern Z (2014). Non-high-density lipoprotein cholesterol independently predicts new onset of non-alcoholic fatty liver disease. Liver Int.

[26] Zou Y, Lan J, Zhong Y, Yang S, Zhang H, Xie G (2021). Association of remnant cholesterol with nonalcoholic fatty liver disease: a general population-based study. Lipids Health Dis.

[27] Wu KT, Kuo PL, Su SB, Chen YY, Yeh ML, Huang CI (2016). Nonalcoholic fatty liver disease severity is associated with the ratios of total cholesterol and triglycerides to high-density lipoprotein cholesterol. J Clin Lipidol.

[28] Fan N, Peng L, Xia Z, Zhang L, Song Z, Wang Y (2019). Triglycerides to high-density lipoprotein cholesterol ratio as a surrogate for nonalcoholic fatty liver disease: a cross-sectional study. Lipids Health Dis.

[29] Zou Y, Zhong L, Hu C, Zhong M, Peng N, Sheng G (2021). LDL/HDL cholesterol ratio is associated with new-onset NAFLD in Chinese non-obese people with normal lipids: a 5-year longitudinal cohort study. Lipids Health Dis.

[30] Wang K, Shan S, Zheng H, Zhao X, Chen C, Liu C (2018). Non-HDL-cholesterol to HDL-cholesterol ratio is a better predictor of new-onset non-alcoholic fatty liver disease than non-HDL-cholesterol: a cohort study. Lipids Health Dis.

[31] Zou Y, Hu C, Kuang M, Chai Y (2022). Remnant cholesterol/high-density lipoprotein cholesterol ratio is a new powerful tool for identifying non-alcoholic fatty liver disease. BMC Gastroenterol.

[32] Hamaguchi M, Kojima T, Itoh Y, Harano Y, Fujii K, Nakajima T (2007). The severity of ultrasonographic findings in nonalcoholic fatty liver disease reflects the metabolic syndrome and visceral fat accumulation. Am J Gastroenterol.

[33] Sato T, Matsuyama Y (2003). Marginal structural models as a tool for standardization. Epidemiology.

[34] Muanda FT, Weir MA, Bathini L, Blake PG, Chauvin K, Dixon SN (2019). Association of Baclofen with encephalopathy in patients with chronic kidney disease. JAMA.

[35] Kim JH (2019). Multicollinearity and misleading statistical results. Korean J Anesthesiol.

[36] Fitchett EJA, Seale AC, Vergnano S, Sharland M, Heath PT, Saha SK (2016). Strengthening the Reporting of Observational Studies in Epidemiology for Newborn Infection (STROBE-NI): an extension of the STROBE statement for neonatal infection research. Lancet Infect Dis.

[37] VanderWeele TJ (2016). Mediation analysis: a practitioner's guide. Annu Rev Public Health.

[38] Huang TD, Behary J, Zekry A (2020). Non-alcoholic fatty liver disease: a review of epidemiology, risk factors, diagnosis and management. Intern Med J.

[39] Åberg F, Färkkilä M (2020). Drinking and obesity: alcoholic liver disease/nonalcoholic fatty liver disease interactions. Semin Liver Dis.

[40] Hamabe A, Uto H, Imamura Y, Kusano K, Mawatari S, Kumagai K (2011). Impact of cigarette smoking on onset of nonalcoholic fatty liver disease over a 10-year period. J Gastroenterol.

[41] Vekic J, Zeljkovic A, Stefanovic A, Jelic-Ivanovic Z, Spasojevic-Kalimanovska V (2019). Obesity and dyslipidemia. Metabolism.

[42] Imai K, Keele L, Tingley D, Yamamoto T (2010). Causal mediation analysis using R. Lecture Notes Statist.

[43] WHO Expert Consultation (2004). Appropriate body-mass index for Asian populations and its implications for policy and intervention strategies. Lancet.

[44] Zhang QQ, Lu LG (2015). Nonalcoholic fatty liver disease: dyslipidemia, risk for cardiovascular complications, and treatment strategy. J Clin Transl Hepatol.

[45] Eslam M, Newsome PN, Sarin SK, Anstee QM, Targher G, Romero-Gomez M (2020). A new definition for metabolic dysfunction-associated fatty liver disease: an international expert consensus statement. J Hepatol.

[46] Wong VW, Wong GL, Chan RS, Shu SS, Cheung BH, Li LS (2018). Beneficial effects of lifestyle intervention in non-obese patients with non-alcoholic fatty liver disease. J Hepatol.

[47] Quirós-Tejeira RE, Rivera CA, Ziba TT, Mehta N, Smith CW, Butte NF (2007). Risk for nonalcoholic fatty liver disease in Hispanic youth with BMI > or =95th percentile. J Pediatr Gastroenterol Nutr.

[48] Chang Y, Jung HS, Cho J, Zhang Y, Yun KE, Lazo M (2016). Metabolically healthy obesity and the development of nonalcoholic fatty liver disease. Am J Gastroenterol.

[49] Liu M, Wang J, Zeng J, Cao X, He Y (2017). Association of NAFLD with diabetes and the impact of BMI changes: a 5-year cohort study based on 18,507 elderly. J Clin Endocrinol Metab.

[50] Rong Y, Chun-Yan N, Hong-Xin Z, Lu Y, Wen W, Yu T (2018). Association of adolescent obesity with nonalcoholic fatty liver disease and related risk factors in Xi'an, China. Ann Hepatol.

[51] Ballestri S, Nascimbeni F, Romagnoli D, Lonardo A (2016). The independent predictors of non-alcoholic steatohepatitis and its individual histological features: insulin resistance, serum uric acid, metabolic syndrome, alanine aminotransferase and serum total cholesterol are a clue to pathogenesis and candidate targets for treatment. Hepatol Res.

[52] McCullough A, Previs SF, Dasarathy J, Lee K, Osme A, Kim C (2019). HDL flux is higher in patients with nonalcoholic fatty liver disease. Am J Physiol Endocrinol Metab.

[53] Xing J, Guan X, Zhang Q, Chen S, Wu S, Sun X (2021). Triglycerides mediate body mass index and nonalcoholic fatty liver disease: a population-based study. Obes Facts.

[54] Liu T, Liu S (2020). The impacts of coal dust on miners' health: a review. Environ Res.

[55] Fomenko DV, Gorokhova LG, Panev NI, Kazitskaia AS, Bondarev OI. Clinical and experimental studies of metabolic response to chronic exposure to coal dust. Med Tr Prom Ekol. 2011;15–21. 21506373

[56] Conklin DJ, Barski OA, Lesgards JF, Juvan P, Rezen T, Rozman D (2010). Acrolein consumption induces systemic dyslipidemia and lipoprotein modification. Toxicol Appl Pharmacol.

[57] Peng F, Dai J, Qian Q, Cao X, Wang L, Zhu M, et al. Serum metabolic profiling of coal worker's pneumoconiosis using untargeted lipidomics. Environ Sci Pollut Res Int. 2022. 10.1007/s11356-022-21905-4 35796929

[58] Gasparotto J, Chaves PR, da Boit MK, da Rosa-Siva HT, Bortolin RC, Silva LFO (2018). Obese rats are more vulnerable to inflammation, genotoxicity and oxidative stress induced by coal dust inhalation than non-obese rats. Ecotoxicol Environ Saf.

[59] Chen L, Fan Z, Sun X, Qiu W, Mu W, Chai K (2022). Diet-derived antioxidants and nonalcoholic fatty liver disease: a Mendelian randomization study. Hepatol Int.

[60] Foster T, Budoff MJ, Saab S, Ahmadi N, Gordon C, Guerci AD (2011). Atorvastatin and antioxidants for the treatment of nonalcoholic fatty liver disease: the St Francis Heart Study randomized clinical trial. Am J Gastroenterol.

[61] Salomone F, Godos J, Zelber-Sagi S (2016). Natural antioxidants for non-alcoholic fatty liver disease: molecular targets and clinical perspectives. Liver Int.

[62] Chen G, Ni Y, Nagata N, Xu L, Ota T (2016). Micronutrient antioxidants and nonalcoholic fatty liver disease. Int J Mol Sci.

[63] Hernández-Guerra M, Hadjihambi A, Jalan R (2019). Gap junctions in liver disease: implications for pathogenesis and therapy. J Hepatol.

[64] Lu S, Kuang M, Yue J, Hu C, Sheng G, Zou Y (2022). Utility of traditional and non-traditional lipid indicators in the diagnosis of nonalcoholic fatty liver disease in a Japanese population. Lipids Health Dis.

[65] Beyene HB, Olshansky G, Smith AAT, Giles C, Huynh K, Cinel M (2020). High-coverage plasma lipidomics reveals novel sex-specific lipidomic fingerprints of age and BMI: evidence from two large population cohort studies. PLoS Biol.

[66] Ryan DH, Yockey SR (2017). Weight loss and improvement in comorbidity: differences at 5%, 10%, 15%, and Over. Curr Obes Rep.

[67] Promrat K, Kleiner DE, Niemeier HM, Jackvony E, Kearns M, Wands JR (2010). Randomized controlled trial testing the effects of weight loss on nonalcoholic steatohepatitis. Hepatology.

[68] Lazo M, Solga SF, Horska A, Bonekamp S, Diehl AM, Brancati FL (2010). Effect of a 12-month intensive lifestyle intervention on hepatic steatosis in adults with type 2 diabetes. Diabetes Care.

