# Supplementary material for: Lipid metabolism, BMI and the risk of nonalcoholic fatty liver disease in the general population: evidence from a mediation analysis
Source: J Transl Med. 2023 Mar 13;21:192. doi: 10.1186/s12967-023-04047-0 (PMC10012451; doi:10.1186/s12967-023-04047-0)
Supplement: Supplementary file 1 — Additional file 1: Table S1. Collinearity diagnostics steps of TC with other covariates. Table S2. Collinearity diagnostics steps of HDL-C with other covariates. Table S3. Collinearity diagnostics steps of TG with other covariates. Table S4. Collinearity diagnostics steps of LDL-C with other covariates. Table S5. Collinearity diagnostics steps of non-HDL-C with other covariates. Table S6. Collinearity diagnostics steps of RC with other covariates. Table S7. Collinearity diagnostics steps of TC/HDL-C ratio with other covariates. Table S8. Collinearity diagnostics steps of TG/HDL-C ratio with other covariates. Table S9. Collinearity diagnostics steps of LDL/HDL-C ratio with other covariates. Table S10. Collinearity diagnostics steps of non-HDL/HDL-C ratio with other covariates. Table S11. Collinearity diagnostics steps of RC/HDL-C ratio with other covariates. Table S12. Collinearity diagnostics steps of BMI with other covariates. Table S13. Association of BMI with lipid parameters. Table S14. Mediation analysis for BMI and NAFLD via lipid parameters in the whole population stratified by age. Table S15. Mediation analysis for BMI and NAFLD via lipid parameters in the whole population stratified by BMI. [file 12967_2023_4047_MOESM1_ESM.docx]

Table S1: Collinearity diagnostics steps of TC with other covariates.

|  | Variance inflation factor |
| --- | --- |
|  | Step 1 |
| TC | 1.2 |
| Sex | 3.1 |
| Age | 1.4 |
| Height | 3.5 |
| Weight | 8.7 |
| WC | 5.7 |
| ALT | 4 |
| AST | 3.3 |
| GGT | 1.4 |
| FPG | 1.5 |
| HbA1c | 1.2 |
| SBP | 5.5 |
| DBP | 5.5 |
| Habit of exercise | 1 |
| Drinking status | 1.2 |
| Smoking status | 1.4 |

Abbreviations as in Table ​1.

Note: Variance inflation factor = 1/(1-R^2^).

Table S2: Collinearity diagnostics steps of HDL-C with other covariates.

|  | Variance inflation factor |
| --- | --- |
|  | Step 1 |
| HDL-C | 1.5 |
| Sex | 3.3 |
| Age | 1.3 |
| Height | 3.5 |
| Weight | 8.8 |
| WC | 5.8 |
| ALT | 4.1 |
| AST | 3.3 |
| GGT | 1.4 |
| FPG | 1.5 |
| HbA1c | 1.2 |
| SBP | 5.5 |
| DBP | 5.5 |
| Habit of exercise | 1 |
| Drinking status | 1.2 |
| Smoking status | 1.4 |

Abbreviations as in Table ​1.

Note: Variance inflation factor = 1/(1-R^2^).

Table S3: Collinearity diagnostics steps of TG with other covariates.

|  | Variance inflation factor |
| --- | --- |
|  | Step 1 |
| TG | 1.4 |
| Sex | 3.2 |
| Age | 1.3 |
| Height | 3.5 |
| Weight | 8.7 |
| WC | 5.7 |
| ALT | 4.1 |
| AST | 3.3 |
| GGT | 1.4 |
| FPG | 1.5 |
| HbA1c | 1.2 |
| SBP | 5.5 |
| DBP | 5.6 |
| Habit of exercise | 1 |
| Drinking status | 1.2 |
| Smoking status | 1.4 |

Abbreviations as in Table ​1.

Note: Variance inflation factor = 1/(1-R^2^).

Table S4: Collinearity diagnostics steps of LDL-C with other covariates.

|  | Variance inflation factor |
| --- | --- |
|  | Step 1 |
| LDL-C | 1.3 |
| Sex | 3.1 |
| Age | 1.4 |
| Height | 3.6 |
| Weight | 8.7 |
| WC | 5.7 |
| ALT | 4.1 |
| AST | 3.3 |
| GGT | 1.4 |
| FPG | 1.5 |
| HbA1c | 1.2 |
| SBP | 5.5 |
| DBP | 5.6 |
| Habit of exercise | 1 |
| Drinking status | 1.2 |
| Smoking status | 1.4 |

Abbreviations as in Table ​1.

Note: Variance inflation factor = 1/(1-R^2^).

Table S5: Collinearity diagnostics steps of non-HDL-C with other covariates.

|  | Variance inflation factor |
| --- | --- |
|  | Step 1 |
| Non-HDL-C | 1.4 |
| Sex | 3.2 |
| Age | 1.4 |
| Height | 3.6 |
| Weight | 8.8 |
| WC | 5.7 |
| ALT | 4.1 |
| AST | 3.3 |
| GGT | 1.4 |
| FPG | 1.5 |
| HbA1c | 1.2 |
| SBP | 5.5 |
| DBP | 5.6 |
| Habit of exercise | 1 |
| Drinking status | 1.2 |
| Smoking status | 1.4 |

Abbreviations as in Table ​1.

Note: Variance inflation factor = 1/(1-R^2^).

Table S6: Collinearity diagnostics steps of RC with other covariates.

|  | Variance inflation factor |
| --- | --- |
|  | Step 1 |
| RC | 1.5 |
| Sex | 3.2 |
| Age | 1.4 |
| Height | 3.6 |
| Weight | 8.8 |
| WC | 5.7 |
| ALT | 4.1 |
| AST | 3.3 |
| GGT | 1.4 |
| FPG | 1.5 |
| HbA1c | 1.2 |
| SBP | 5.5 |
| DBP | 5.6 |
| Habit of exercise | 1 |
| Drinking status | 1.2 |
| Smoking status | 1.4 |

Abbreviations as in Table ​1.

Note: Variance inflation factor = 1/(1-R^2^).

Table S7: Collinearity diagnostics steps of TC/HDL-C ratio with other covariates.

|  | Variance inflation factor |
| --- | --- |
|  | Step 1 |
| TC/HDL-C ratio | 1.6 |
| Sex | 3.2 |
| Age | 1.3 |
| Height | 3.6 |
| Weight | 8.8 |
| WC | 5.8 |
| ALT | 4.1 |
| AST | 3.3 |
| GGT | 1.4 |
| FPG | 1.5 |
| HbA1c | 1.2 |
| SBP | 5.5 |
| DBP | 5.6 |
| Habit of exercise | 1 |
| Drinking status | 1.2 |
| Smoking status | 1.4 |

Abbreviations as in Table ​1.

Note: Variance inflation factor = 1/(1-R^2^).

Table S8: Collinearity diagnostics steps of TG/HDL-C ratio with other covariates.

|  | Variance inflation factor |
| --- | --- |
|  | Step 1 |
| TG/HDL-C ratio | 1.4 |
| Sex | 3.2 |
| Age | 1.3 |
| Height | 3.5 |
| Weight | 8.8 |
| WC | 5.7 |
| ALT | 4.1 |
| AST | 3.3 |
| GGT | 1.4 |
| FPG | 1.5 |
| HbA1c | 1.2 |
| SBP | 5.5 |
| DBP | 5.6 |
| Habit of exercise | 1 |
| Drinking status | 1.2 |
| Smoking status | 1.4 |

Abbreviations as in Table ​1.

Note: Variance inflation factor = 1/(1-R^2^).

Table S9: Collinearity diagnostics steps of LDL/HDL-C ratio with other covariates.

|  | Variance inflation factor |
| --- | --- |
|  | Step 1 |
| LDL-C/HDL-C ratio | 1.6 |
| Sex | 3.2 |
| Age | 1.3 |
| Height | 3.6 |
| Weight | 8.8 |
| WC | 5.8 |
| ALT | 4.1 |
| AST | 3.3 |
| GGT | 1.4 |
| FPG | 1.5 |
| HbA1c | 1.2 |
| SBP | 5.5 |
| DBP | 5.6 |
| Habit of exercise | 1 |
| Drinking status | 1.2 |
| Smoking status | 1.4 |

Abbreviations as in Table ​1.

Note: Variance inflation factor = 1/(1-R^2^).

Table S10: Collinearity diagnostics steps of non-HDL/HDL-C ratio with other covariates.

|  | Variance inflation factor |
| --- | --- |
|  | Step 1 |
| Non-HDL-C/HDL-C ratio | 1.6 |
| Sex | 3.2 |
| Age | 1.3 |
| Height | 3.6 |
| Weight | 8.8 |
| WC | 5.8 |
| ALT | 4.1 |
| AST | 3.3 |
| GGT | 1.4 |
| FPG | 1.5 |
| HbA1c | 1.2 |
| SBP | 5.5 |
| DBP | 5.6 |
| Habit of exercise | 1 |
| Drinking status | 1.2 |
| Smoking status | 1.4 |

Abbreviations as in Table ​1.

Note: Variance inflation factor = 1/(1-R^2^).

Table S11: Collinearity diagnostics steps of RC/HDL-C ratio with other covariates.

|  | Variance inflation factor |
| --- | --- |
|  | Step 1 |
| RC/HDL-C ratio | 1.6 |
| Sex | 3.2 |
| Age | 1.3 |
| Height | 3.6 |
| Weight | 8.8 |
| WC | 5.7 |
| ALT | 4.1 |
| AST | 3.3 |
| GGT | 1.4 |
| FPG | 1.5 |
| HbA1c | 1.2 |
| SBP | 5.5 |
| DBP | 5.6 |
| Habit of exercise | 1 |
| Drinking status | 1.2 |
| Smoking status | 1.4 |

Abbreviations as in Table ​1.

Note: Variance inflation factor = 1/(1-R^2^).

Table S12: Collinearity diagnostics steps of BMI with other covariates.

|  | Variance inflation factor | |
| --- | --- | --- |
|  | Step 1 | Step 2 |
| BMI | 95.4 | 5 |
| Sex | 3.2 | 3.1 |
| Age | 1.3 | 1.3 |
| Height | 51.7 | 2.8 |
| Weight | 167.9 | NA |
| WC | 5.9 | 5.9 |
| ALT | 4 | 4 |
| AST | 3.3 | 3.3 |
| GGT | 1.4 | 1.4 |
| FPG | 1.5 | 1.5 |
| HbA1c | 1.2 | 1.2 |
| SBP | 5.5 | 5.5 |
| DBP | 5.5 | 5.5 |
| Habit of exercise | 1 | 1 |
| Drinking status | 1.2 | 1.2 |
| Smoking status | 1.4 | 1.4 |

Abbreviations as in Table ​1.

Note: Variance inflation factor = 1/(1-R^2^).

Table S13: Association of BMI with lipid parameters.

|  | *β*（95%CI） | |  |
| --- | --- | --- | --- |
|  | Non-adjusted | Non-adjusted | *P*-value |
| TC | 0.71 (0.65, 0.77) | 0.14 (0.11, 0.17) | ＜0.001 |
| TG | 1.97 (1.90, 2.05) | 0.24 (0.21, 0.28) | ＜0.001 |
| HDL-C | -3.23 (-3.35, -3.12) | -0.45 (-0.53, -0.38) | ＜0.001 |
| LDL-C | 1.36 (1.30, 1.43) | 0.24 (0.21, 0.28) | ＜0.001 |
| Non-HDL-C | 1.28 (1.23, 1.34) | 0.22 (0.19, 0.26) | ＜0.001 |
| RC | 6.69 (6.46, 6.91) | 1.03 (0.88, 1.17) | ＜0.001 |
| TC/HDL-C ratio | 1.26 (1.22, 1.30) | 0.21 (0.19, 0.24) | ＜0.001 |
| TG/HDL-C ratio | 1.69 (1.63, 1.75) | 0.21 (0.17, 0.25) | ＜0.001 |
| LDL-C/HDL-C ratio | 1.53 (1.49, 1.58) | 0.26 (0.23, 0.29) | ＜0.001 |
| Non-HDL-C/HDL-C ratio | 1.26 (1.22, 1.30) | 0.21 (0.19, 0.24) | ＜0.001 |
| RC/HDL-C ratio | 5.40 (5.23, 5.57) | 0.80 (0.69, 0.92) | ＜0.001 |

Adjusting variables: sex, age, WC, SBP, DBP, FPG, HbA1c, habit of exercise, smoking status and drinking status.

Table S14: Mediation analysis for BMI and NAFLD via lipid parameters in the whole population stratified by age.

| Mediator | Total effect | Mediation effect | Direct effect | PM(%) | p-value of PM |
| --- | --- | --- | --- | --- | --- |
| **Age: ＜30years old (n=401)** | | | | | |
| TC | 0.037 (-0.002, 0.077) | 0.001 (-0.004, 0.005) | 0.036 (-0.003, 0.076) | - | 0.768 |
| TG | 0.037 (-0.002, 0.078) | 0.008 (0.000, 0.017) | 0.029 (-0.010, 0.069) | - | 0.096 |
| HDL-C | 0.037 (-0.003, 0.078) | 0.004 (-0.002, 0.013) | 0.033 (-0.005, 0.073) | - | 0.294 |
| LDL-C | 0.037 (-0.003, 0.078) | 0.004 (-0.001, 0.010) | 0.033 (-0.007, 0.074) | - | 0.216 |
| Non-HDL-C | 0.037 (-0.003, 0.078) | 0.005 (-0.000, 0.012) | 0.032 (-0.008, 0.072) | - | 0.124 |
| RC | 0.037 (-0.002, 0.078) | 0.010 (0.002, 0.019) | 0.027 (-0.013, 0.067) | - | 0.076 |
| TC/HDL-C ratio | 0.037 (-0.002, 0.078) | 0.011 (0.002, 0.022) | 0.026 (-0.013, 0.065) | - | 0.088 |
| TG/HDL-C ratio | 0.037 (-0.002, 0.078) | 0.010 (0.000, 0.022) | 0.027 (-0.012, 0.066) | - | 0.106 |
| LDL/HDL-C ratio | 0.037 (-0.002, 0.078) | 0.010 (0.001, 0.020) | 0.027 (-0.012, 0.067) | - | 0.094 |
| Non-HDL-C/HDL-C ratio | 0.037 (-0.002, 0.078) | 0.011 (0.002, 0.022) | 0.026 (-0.012, 0.065) | - | 0.090 |
| RC/HDL-C ratio | 0.037 (-0.002, 0.077) | 0.013 (0.003, 0.026) | 0.023 (-0.015, 0.063) | - | 0.078 |
| **Age: 30-44 years old (n=7,901)** | | | | | |
| TC | 0.119 (0.102, 0.137) | 0.003 (0.001, 0.004) | 0.116 (0.099, 0.134) | 2.4 | ＜0.001 |
| TG | 0.119 (0.102, 0.137) | 0.012 (0.008, 0.015) | 0.107 (0.090, 0.125) | 9.8 | ＜0.001 |
| HDL-C | 0.119 (0.102, 0.137) | 0.005 (0.003, 0.008) | 0.114 (0.097, 0.132) | 4.4 | ＜0.001 |
| LDL-C | 0.119 (0.102, 0.137) | 0.005 (0.003, 0.007) | 0.114 (0.097, 0.132) | 3.8 | ＜0.001 |
| Non-HDL-C | 0.119 (0.102, 0.137) | 0.008 (0.005, 0.010) | 0.111 (0.094, 0.129) | 6.3 | ＜0.001 |
| RC | 0.119 (0.102, 0.137) | 0.015 (0.011, 0.019) | 0.104 (0.087, 0.121) | 12.7 | ＜0.001 |
| TC/HDL-C ratio | 0.119 (0.102, 0.137) | 0.013 (0.010, 0.016) | 0.106 (0.089, 0.124) | 10.9 | ＜0.001 |
| TG/HDL-C ratio | 0.119 (0.102, 0.137) | 0.010 (0.007, 0.014) | 0.109 (0.092, 0.126) | 8.8 | ＜0.001 |
| LDL/HDL-C ratio | 0.119 (0.102, 0.137) | 0.011 (0.008, 0.014) | 0.108 (0.091, 0.126) | 9.2 | ＜0.001 |
| Non-HDL-C/HDL-C ratio | 0.119 (0.102, 0.137) | 0.013 (0.010, 0.016) | 0.106 (0.089, 0.124) | 10.9 | ＜0.001 |
| RC/HDL-C ratio | 0.119 (0.102, 0.137) | 0.015 (0.011, 0.018) | 0.105 (0.088, 0.122) | 12.2 | ＜0.001 |
| **Age: 45-59 years old (n=3,994)** | | | | | |
| TC | 0.103 (0.080, 0.126) | -0.00 (-0.001, 0.001) | 0.103 (0.080, 0.126) | - | 0.812 |
| TG | 0.103 (0.080, 0.127) | 0.007 (0.003, 0.010) | 0.096 (0.073, 0.118) | 6.6 | ＜0.001 |
| HDL-C | 0.103 (0.080, 0.125) | 0.009 (0.006, 0.013) | 0.094 (0.071, 0.117) | 8.4 | ＜0.001 |
| LDL-C | 0.103 (0.080, 0.126) | 0.001 (-0.001, 0.002) | 0.102 (0.080, 0.125) | - | 0.546 |
| Non-HDL-C | 0.103 (0.080, 0.126) | 0.002 (0.000, 0.004) | 0.101 (0.078, 0.123) | 2.1 | ＜0.001 |
| RC | 0.103 (0.080, 0.126) | 0.008 (0.005, 0.011) | 0.095 (0.072, 0.117) | 7.7 | ＜0.001 |
| TC/HDL-C ratio | 0.103 (0.080, 0.126) | 0.010 (0.007, 0.014) | 0.093 (0.070, 0.115) | 9.8 | ＜0.001 |
| TG/HDL-C ratio | 0.103 (0.080, 0.127) | 0.008 (0.005, 0.012) | 0.095 (0.072, 0.117) | 8 | ＜0.001 |
| LDL/HDL-C ratio | 0.103 (0.080, 0.126) | 0.009 (0.005, 0.012) | 0.094 (0.071, 0.117) | 8.3 | ＜0.001 |
| Non-HDL-C/HDL-C ratio | 0.103 (0.080, 0.126) | 0.010 (0.007, 0.014) | 0.093 (0.070, 0.115) | 9.8 | ＜0.001 |
| RC/HDL-C ratio | 0.069 (0.047, 0.090) | 0.010 (0.006, 0.014) | 0.059 (0.036, 0.081) | 14.1 | ＜0.001 |
| **Age: ≥60 years old (n=635)** | | | | | |
| TC | 0.108 (0.048, 0.171) | 0.001 (-0.003, 0.006) | 0.107 (0.047, 0.169) | - | 0.724 |
| TG | 0.108 (0.047, 0.171) | 0.000 (-0.008, 0.008) | 0.108 (0.049, 0.169) | - | 0.910 |
| HDL-C | 0.108 (0.048, 0.170) | 0.002 (-0.005, 0.009) | 0.107 (0.047, 0.169) | - | 0.580 |
| LDL-C | 0.108 (0.048, 0.171) | 0.002 (-0.002, 0.008) | 0.106 (0.045, 0.167) | - | 0.386 |
| Non-HDL-C | 0.108 (0.047, 0.171) | 0.003 (-0.001, 0.010) | 0.105 (0.045, 0.166) | - | 0.250 |
| RC | 0.108 (0.047, 0.171) | 0.003 (-0.005, 0.011) | 0.105 (0.046, 0.166) | - | 0.530 |
| TC/HDL-C ratio | 0.108 (0.048, 0.171) | 0.004 (-0.001, 0.011) | 0.104 (0.044, 0.166) | - | 0.188 |
| TG/HDL-C ratio | 0.108 (0.048, 0.171) | 0.002 (-0.005, 0.010) | 0.106 (0.047, 0.167) | - | 0.566 |
| LDL/HDL-C ratio | 0.108 (0.048, 0.170) | 0.003 (-0.002, 0.010) | 0.105 (0.045, 0.166) | - | 0.280 |
| Non-HDL-C/HDL-C ratio | 0.108 (0.049, 0.171) | 0.004 (-0.001, 0.011) | 0.104 (0.044, 0.166) | - | 0.182 |
| RC/HDL-C ratio | 0.108 (0.048, 0.171) | 0.004 (-0.002, 0.012) | 0.104 (0.045, 0.165) | - | 0.240 |

Abbreviations: PM: propotion mediate; other abbreviations as in Table ​1.

Adjusting variables: sex, WC, SBP, DBP, ALT, AST, GGT, FPG, HbA1c, habit of exercise, smoking status and drinking status.

Table S15: Mediation analysis for BMI and NAFLD via lipid parameters in the whole population stratified by BMI.

| Mediator | Total effect | Mediation effect | Direct effect | PM(%) | | p-value  of PM |
| --- | --- | --- | --- | --- | --- | --- |
| **BMI＜24kg/m^2^ (n=10,881)** | | | | | | |
| TC | 0.026 (0.015, 0.037) | 0.001 (0.001, 0.002) | 0.024 (0.013, 0.035) | 5.6 | ＜0.001 | |
| TG | 0.026 (0.015, 0.037) | 0.006 (0.004, 0.008) | 0.020 (0.009, 0.031) | 26.4 | ＜0.001 | |
| HDL-C | 0.026 (0.015, 0.037) | 0.004 (0.003, 0.005) | 0.022 (0.011, 0.033) | 15 | ＜0.001 | |
| LDL-C | 0.026 (0.015, 0.037) | 0.003 (0.002, 0.004) | 0.023 (0.012, 0.034) | 11.8 | ＜0.001 | |
| Non-HDL-C | 0.026 (0.015, 0.037) | 0.005 (0.003, 0.006) | 0.021 (0.010, 0.032) | 18 | ＜0.001 | |
| RC | 0.026 (0.015, 0.037) | 0.009 (0.007, 0.011) | 0.017 (0.006, 0.028) | 33.4 | ＜0.001 | |
| TC/HDL-C ratio | 0.026 (0.015, 0.037) | 0.008 (0.006, 0.010) | 0.018 (0.007, 0.029) | 30.4 | ＜0.001 | |
| TG/HDL-C ratio | 0.026 (0.015, 0.037) | 0.006 (0.004, 0.007) | 0.020 (0.010, 0.031) | 21.5 | ＜0.001 | |
| LDL/HDL-C ratio | 0.026 (0.015, 0.037) | 0.007 (0.005, 0.009) | 0.019 (0.008, 0.030) | 26.7 | ＜0.001 | |
| Non-HDL-C/HDL-C ratio | 0.026 (0.015, 0.037) | 0.008 (0.006, 0.010) | 0.018 (0.007, 0.029) | 30.5 | ＜0.001 | |
| RC/HDL-C ratio | 0.026 (0.015, 0.037) | 0.008 (0.006, 0.010) | 0.017 (0.007, 0.029) | 32.1 | ＜0.001 | |
| **BMI≥ 24(n=3,370)** | | | | | | |
| TC | 0.071 (0.043, 0.099) | 0.000 (-0.001, 0.002) | 0.071 (0.043, 0.098) | - | 0.406 | |
| TG | 0.071 (0.043, 0.100) | 0.002 (-0.003, 0.007) | 0.069 (0.041, 0.096) | - | 0.432 | |
| HDL-C | 0.071 (0.042, 0.099) | 0.005 (0.001, 0.009) | 0.066 (0.038, 0.094) | 6.4 | 0.016 | |
| LDL-C | 0.071 (0.042, 0.099) | 0.001 (-0.000, 0.003) | 0.070 (0.042, 0.098) | - | 0.160 | |
| Non-HDL-C | 0.071 (0.043, 0.100) | 0.002 (-0.000, 0.004) | 0.069 (0.041, 0.097) | - | 0.128 | |
| RC | 0.071 (0.043, 0.100) | 0.003 (-0.002, 0.008) | 0.068 (0.040, 0.095) | - | 0.216 | |
| TC/HDL-C ratio | 0.071 (0.042, 0.100) | 0.004 (-0.000, 0.008) | 0.067 (0.039, 0.095) | - | 0.058 | |
| TG/HDL-C ratio | 0.071 (0.043, 0.100) | 0.002 (-0.003, 0.007) | 0.069 (0.040, 0.096) | - | 0.356 | |
| LDL/HDL-C ratio | 0.071 (0.042, 0.099) | 0.003 (-0.000, 0.007) | 0.068 (0.039, 0.095) | - | 0.054 | |
| Non-HDL-C/HDL-C ratio | 0.071 (0.042, 0.100) | 0.004 (-0.000, 0.008) | 0.067 (0.039, 0.095) | - | 0.058 | |
| RC/HDL-C ratio | 0.071 (0.043, 0.100) | 0.004 (-0.001, 0.008) | 0.067 (0.039, 0.095) | - | 0.166 | |

Abbreviations: PM: propotion mediate; other abbreviations as in Table ​1.

Adjusting variables: sex, Age, WC, SBP, DBP, ALT, AST, GGT, FPG, HbA1c, habit of exercise, smoking status and drinking status.
